# Effect of Hydration on Pulmonary Function and Development of Exercise-Induced Bronchoconstriction among Professional Male Cyclists

**DOI:** 10.3390/arm91030019

**Published:** 2023-06-07

**Authors:** Konstantinos M. Pigakis, Vasileios T. Stavrou, Ioannis Pantazopoulos, Zoe Daniil, Aggeliki K. Kontopodi-Pigaki, Konstantinos Gourgoulianis

**Affiliations:** 1 Department of Respiratory & Critical Care Medicine, Creta Interclinic, 71304 Heraklion, Greece; 2 Laboratory of Cardiopulmonary Testing and Pulmonary Rehabilitation, Department of Respiratory Medicine, Faculty of Medicine, University of Thessaly, 41110 Larissa, Greece; 3 Emergency Medicine, Faculty of Medicine, University of Thessaly, 41110 Larissa, Greece

**Keywords:** exercise-induced bronchoconstriction, elite athletes, pulmonary function tests, hydration, exercise-induced dehydration, exercise-induced airway injury

## Abstract

**Highlights:**

**What are the main findings?**

- Exercise-induced bronchoconstriction (EIB) is a common problem in elite athletes. This study aimed to investigate the effects of systemic hydration on pulmonary function and to establish whether it can reverse dehydration-induced alterations in pulmonary function.
- Systemic hydration had a positive effect on both pulmonary function and exercise capacity (VO_2_ max).

**What are the implications of the main findings?**

- Hydration potentially plays a regulatory role in stabilizing the airway in elite athletes, protecting them from airway hyper-responsiveness.
- Of particular interest are the small airways, which appear to be affected independently or in combination with a decrease in FEV1 and proper hydration can protect them from further injury.

**Abstract:**

Background: Exercise-induced bronchoconstriction (EIB) is a common problem in elite athletes. Classical pathways in the development of EIB include the osmotic and thermal theory as well as the presence of epithelial injury in the airway, with local water loss being the main trigger of EIB. This study aimed to investigate the effects of systemic hydration on pulmonary function and to establish whether it can reverse dehydration-induced alterations in pulmonary function. Materials and Methods: This follow-up study was performed among professional cyclists, without a history of asthma and/or atopy. Anthropometric characteristics were recorded for all participants, and the training age was determined. In addition, pulmonary function tests and specific markers such as fractional exhaled nitric oxide (FeNO) and immunoglobulin E (IgE) were measured. All the athletes underwent body composition analysis and cardiopulmonary exercise testing (CPET). After CPET, spirometry was followed at the 3rd, 5th, 10th, 15th, and 30th min. This study was divided into two phases: before and after hydration. Cyclists, who experienced a decrease in Forced Expiratory Volume in one second (FEV_1_) ≥ 10% and/or Maximal Mild-Expiratory Flow Rate (MEF_25–75_) ≥ 20% after CPET in relation to the results of the spirometry before CPET, repeated the test in 15-20 days, following instructions for hydration. Results: One hundred male cyclists (*n* = 100) participated in Phase A. After exercise, there was a decrease in all spirometric parameters (*p* < 0.001). In Phase B, after hydration, in all comparisons, the changes in spirometric values were significantly lower than those in Phase A (*p* < 0.001). Conclusions: The findings of this study suggest that professional cyclists have non-beneficial effects on respiratory function. Additionally, we found that systemic hydration has a positive effect on spirometry in cyclists. Of particular interest are small airways, which appear to be affected independently or in combination with the decrease in FEV_1_. Our data suggest that pulmonary function improves systemic after hydration.

## 1. Introduction

### 1.1. Exercise-Induced Bronchoconstriction

Exercise-induced bronchoconstriction (EIB) is defined as the transient airway contraction after intense exercise without a history of bronchial asthma [1,2]. Exercise is a common EIB trigger in elite athletes and can lead to potential limitations in their performance [2,3,4,5,6]. Exercise-induced bronchoconstriction in athletes has unusual clinical characteristics; therefore, some investigators believe that it is a different asthma phenotype [6]. Many elite athletes with EIB have no history of asthma, suggesting that environmental factors are independently more significant than genetic factors in these cases. Environmental factors may have an additional effect on the genetic predisposition to develop bronchoconstriction as well as being independent and significant etiological factors [2]. Many specialists have proposed the designation of EIB with asthma (EIB_A_—bronchoconstriction after exercise in asthmatic athletes) and EIB without asthma (EIB_WA_—bronchoconstriction triggered by exercise in athletes without other symptoms of asthma) [2,7]. For the aims of this study, EIB_WA_ is referred to as EIB. The prevalence of EIB is 20% to 50% in elite athletes, especially those engaged in high-intensity aerobic exercise [2,8]. The main stimuli for the appearance of EIB are hyperventilation [9] and a hyperosmotic environment on the airway surface [10]. In elite athletes, extreme ventilation conditions cause airway epithelial injuries with the release of special mediators that trigger EIB [1,11,12,13]. Some studies suggest that long-term and high-intensity training in endurance sports may not only stimulate EIB but also the subsequent appearance of bronchial hyperreactivity outside exercise conditions or permanent airway remodeling [14,15,16]. The epithelium of small airways seems to be the most affected by injury and repair, as shown in mouse studies [6].

### 1.2. Hydration and Pulmonary Function

Systemic dehydration commonly occurs in athletes who perform sustained physical activity [17]. Even at mild levels (i.e., 2–3% body mass loss [18]), systemic dehydration can have unfavorable effects on multiple organ systems [19]. Limited and contradictory data currently exist regarding the effects of systemic dehydration on the respiratory system and the development of EIB in elite athletes. Previous studies showed harmful alterations in expiratory flow or lung volume in healthy populations [20] and in athletes with asthma [21] following mild systemic dehydration. However, there is another study [22] which showed improvements in pulmonary function in healthy adults following moderate dehydration (4.5% body mass loss). 

The fluid supply to the airways stems primarily from bronchial circulation [23]. Optimal lung fluid balance is a critical component of normal pulmonary function [24], with bronchial tree surface liquid dehydration implicated in several respiratory diseases, such as EIB [25]. Water flows across bronchial epithelium in response to osmotic gradient. Whole-body dehydration leads to bronchial blood flow and/or compositional changes, which may compromise airway hydration. Alterations in airway surface liquid thickness, composition, and/or rheology can promote airway instability and provoke premature airway closure [26].

### 1.3. Cycling

Cycling is recognized as one of the most challenging endurance sporting events worldwide [27,28]. Competitive cycling is highly stressful for both aerobic and anaerobic metabolisms. A professional cyclist can be defined as a cyclist who performs high training volumes (~32,500 km) during the competitive season, which includes 90–100 race days [27,28]. Indeed, professional cyclists are often considered to represent the «elite» and typically demonstrate advanced cardiorespiratory capacity [27,28]. It is now recognized that cycling places significant demands on the respiratory system [29,30] during periods of maximal intensity exercise, which can elicit ventilation rates > 150 L/min [31]. In addition to the stress of sustained hyperventilation, elite cyclists are regularly exposed to variable environmental conditions (i.e., fluctuations in temperature and humidity), aeroallergens, and particles resulting from the combustion that occurs in the engines. It has been argued that this type of endurance exercise may result in airway changes [10] and has been recognized to occur in up to one in five elite endurance athletes and is prominent in competitive cyclists [32,33].

Many professional cyclists often report a heightened perception of breathlessness, end-race cough, and the sensation that they have «smaller lungs» during or following cycling competition. One study supports the deterioration in lung function and presents troublesome respiratory symptoms following endurance sporting events [34]. This study aimed to examine evidence of EIB in triathletes and assess whether changes in FEV_1_ were related to respiratory symptoms, training volume, and race time. Lung function was measured before the race, 8–10 min after the race (post-test 1), and the day after the race (post-test 2). Respiratory symptoms and training volume were recorded using a questionnaire. Twenty-six participants (46%) presented with EIB at post-test 1 and 16 (28%) at post-test 2. The lung function variables were significantly reduced from baseline to post-tests 1 and 2. Changes in FEV_1_ did not correlate with weekly training hours or race. In addition, a weak correlation was observed between the maximal reduction in FEV_1_ and respiratory symptoms, which may affect athletic performance and limit exercise tolerance. 

In line with the findings of a study in asthmatic athletes [21,35], we hypothesized that during or immediately after exercise, pulmonary function would be affected in professional cyclists without asthma and that whole-body hydration can improve and restore lung function. This study aimed to investigate the effects of whole-body hydration on pulmonary function and to establish whether it can reverse dehydration-induced alterations in pulmonary function and the development of EIB in professional cyclists.

## 2. Materials and Methods 

One hundred professional male cyclists (*n* = 100) were invited to participate in this follow-up study (cohort study). All cyclists were accustomed to cycling for more than 15 h per week in sessions lasting at least 3 to 4 h. They were asked not to drink caffeine or consume alcohol during the sessions of the study, not to exercise 48 h before a session, and arrive at the laboratory between 9:00 am and 2:00 pm. The laboratory has a portable air purification system (Health-Way Deluxe, DFS Technology—VOC Filter, New York, NY, USA). The study protocol was approved by the ethics committee of Creta Inter Clinic General Hospital (Registry number: 129/16-09-2020). All participants provided written informed consent in accordance with the Helsinki Declaration and personal data according to the European Parliament and Council of the European Union.

This study took place from October 2020 to January 2021. The Inclusion criteria were as follows: absence of a history of bronchial asthma as evidenced by the medical history and pulmonary function tests, normal heart ultrasound, and the training age (>3 years) in the sport. Exclusion criteria were: Female professional cyclists, COVID-19 infection, age (18 < age > 35 years), smoking habits, craniofacial and upper airway deformities (high probability of misapplication of the ergospirometry face mask), having an injury for the last 12 months, anemia (Hb < 13.5 g/dL), upper and lower respiratory system seasonal allergies, high levels of FeNO (>25 ppb), and high levels of IgE (>100 UI/mL).

The cause of female exclusion from the study is the known physiological differences between the sexes, specifically regarding reproductive endocrinology, menstrual cycle, and hormonal contraceptive use. 

### 2.1. Procedures 

A detailed history of known medical conditions, current medications, and smoking habits was routinely obtained from all athletes. All cyclists were free from respiratory tract infection two weeks before study entry. In addition, a clinical examination was performed on all participants, which included the following: recording of demographic (sex, age) and anthropometric characteristics such as height, body mass, and Body Mass Index (BMI) [36,37,38], and determination of training age. Pulmonary function was assessed [39], and specific markers such as FeNO and IgE, were measured. Finally, the athletes underwent body composition analysis [38], and a cardiopulmonary exercise test (CPET) [40]. With the completion of CPET, spirometry was followed at the 3rd, 5th,10th, 15th, and 30th min. Athletes, who experienced a decrease in FEV_1_ and/or MEF_25–75_ in spirometry after CPET in relation to the results of the spirometry before CPET, re-underwent in 15–20 days, following instructions for fluid intake, and the results were correlated with the hydration level. 

### 2.2. Spirometry 

Lung function was assessed according to the ATS/ERS guidelines [39] on a spirometer (Ergocard Clinical with ExpAir Software, Medisoft Group, Namur, Belgium), and the following parameters were recorded: Forced Vital Capacity (FVC), FEV_1_, and MEF_25–75_. Spirometry was performed in a quiet and comfortable environment. Measurements were conducted by the same operator, in the morning. The subject was seated, with shoulders slightly back and, the chin slightly elevated, and a nose clip was used. There were four distinct phases of the FVC maneuver: (1) maximal inspiration, (2) a blast of expiration, (3) continued complete expiration [the expiration stops when a plateau has been reached or the forced expiratory time (FET) reaches 15 s, and (4) inspiration for a maximal flow back to maximum lung volume. The subject inserted a mouthpiece and was instructed to breathe normally or easily. For each pulmonary function test, the best three of all performed measurements, that met the ATS/ERS criteria were evaluated. The largest one was retained to calculate the spirometric values. A decrease in FEV_1_ ≥ 10% and/or MEF_25–75_ ≥ 20% in spirometry was considered a minimal clinically significant difference [8,41]. 

### 2.3. Airway Inflammation 

Airway inflammation was assessed by measuring FeNO using a specific breath analyzer (FeNO Breath Analyzer, Bedfont Scientific Co., Maidstone, UK) and evaluated against established thresholds: normal < 25 ppb, intermediate: 26–50 ppb, high > 50 ppb [42]. All measurements were performed by the same operator in the morning and before spirometry (spirometric maneuvers reduce FeNO levels). The subject was seated comfortably, and a mouthpiece was used. The participant inhaled over 2 to 3 s through the mouth to total lung capacity and then exhaled immediately. The resultant mouthpiece pressure was at least 5 cm H_2_O; therefore, contamination of expiration with nasal FeNO was excluded. A flow rate of 0.05 L/s was chosen for all participants.

### 2.4. Body Hydration Status

Hydration status was assessed by total body water percentage (TBW%) using whole-body bioelectrical impedance analysis (BIA) and changes in plasma osmolality (P_osm_) of the capillary blood. Total body composition was measured with whole-body BIA using four-pole multifrequency equipment (Tanita BC-613S Fat Meter Scale) using a standard technique [43]. The measurement was conducted by the same operator in the morning at temperatures ranging from 24 to 26 °C. Simultaneously, capillary blood samples were collected from the participant’s fingertips to assess P_osm_. The samples were analyzed immediately after collection. P_osm_ was analyzed using freezing point depression osmometry (Advanced^®^ model 3320 micro-osmometer, Norwood, MA, USA). The subjects were free to have their habitual breakfast three hours before the measurement and should not have exercised 24 h before or consumed alcohol 12 h and water 1 h before the measurements. For the purposes of this study, TBW (%) will be referred to as hydration (%).

### 2.5. Anthropometric Characteristics 

Body mass (kg) and height (cm) were measured according to the manual reference for anthropometric standardization [44]. All the subjects wοre light clothing and were barefoot. Body mass index (BMI) was calculated by dividing the body mass (kg) by the squared height (m) [BMI = Body mass_(kg)_/height_(m)_^2^] (Table 1). 

### 2.6. Systemic Hydration Protocol

Athletes performed two exercise sessions: exercise without fluid intake and exercise accompanied by hydration with water. In every session, the participants exercised until exhaustion. During the first session, the protocol for all the participants was exercised without fluid intake. The time elapsed between the exercise sessions was two weeks. During the two weeks, the cyclists were instructed to drink no less than 3 L of fluid daily (without alcohol), regardless of whether they had an easy or hard training session or a recovery day. There was a phone recall to the subjects every 4 days concerning their adherence to the consumption of 3 L daily. During the second session, all participants were hydrated by a standard hydration protocol before exercise, which included ingesting water at room temperature, mixed with 3 gr NaCl in 1 lit H_2_O to improve fluid retention [45]. The participants gradually consumed 300 mL of water 120 min before exercise, 300 mL 60 min before exercise, and another 600 mL 30 min before exercise.

### 2.7. Cardiopulmonary Exercise Test (CPET)

In this study, CPET was performed using a cycloergometer (Ergocard Clinical with ExpAir Software, Medisoft Group, Namur, Belgium). The CPET protocol consisted of the following procedure: it started with a two-minute rest period, followed by one minute of warm-up pedaling against a minimal load of 20 watts, progressively increasing by 50 watts per three minutes. Environmental indoor conditions during the exercise sessions were kept constant, with a temperature of 24–26 °C, and relative humidity of 45–50%. Participants wore sports clothes commonly used in cycling and were free to have their habitual breakfast 3 h before the exercise session. Participants were asked not to exercise 24 h prior to a session and arrive at the laboratory between 9:00 a.m. and 2:00 p.m. On the first visit, they were given 15 min to adapt to the stationary bicycle of the laboratory. The duration of the test was until exhaustion. CPET was conducted under continuous monitoring of heart rate (HR), 12-lead electrocardiogram (ECG), and pulse oxygen saturation (SpO_2_), while systolic and diastolic blood pressure was recorded every two minutes with a cuff sphygmomanometer. Indications for early test termination included myocardial ischemia, complex ventricular premature beats, grade-2 or grade-3 atrioventricular block, a sudden fall in blood pressure level, by more than 20 mmHg, and elevated blood pressure (>220/120 mmHg) [2,46].

### 2.8. Statistical Analysis

Continuous variables were expressed in the form of mean ± Standard Deviation (SD), while the median (m), interquartile ranges (IQS), and range (min–max) were also used in some cases. Categorical variables are expressed as counts and percentages. For continuous variables, data normality was assessed via the Kolmogorov–Smirnov test. Relationships between continuous variables were assessed via Pearson’s R correlation coefficients. A paired sample t-test was applied to evaluate the differences in spirometry before and after exercise. An independent samples t-test was applied to compare the means of two independent samples. Box and Whisker plots and scatterplots were used to graphically represent the data. IBMS SPSS statistics 26.0 (SPSS Inc., version 26, Chicago, 10 IL, USA) was used for statistical analysis, and *p* < 0.05 was considered statistically significant. 

## 3. Results

### 3.1. Phase A of the Study (before Hydration)

One hundred male professional cyclists (100) participated in Phase A of the study. All participants were young cyclists, within a range of 18–34 years old and a mean age of 27 ± 5 years, while the median age was 30 years. The athletic age ranged between 3 and 19 years, and the mean athletic age was 12 ± 5 years. The mean height of athletes was 177 ± 5 cm, ranging from 177 to 187 cm (min–max), and the mean body mass was 74.7 ± 5.2 kg. The BMI of athletes ranged from 21.5–26.8 kg/m^2^ and the mean and median BMI were 23.8 ± 1.4 and 23.6 kg/m^2^, respectively. Most of the participants (85.0%) were in the normal range of BMI (20.0–25.0 kg/m^2^) and only 15 athletes (15.0%) were in the range of overweight (25.0–26.8 kg/m^2^). Body composition in fat and water was expressed as% of body composition and their respective mean values were 11.6 ± 1.0 and 53 ± 7.0 respectively. The P_osm_ ranged from 278 to 288 m_osm_·kg^−1^ and the mean and median P_osm_ were 283 ± 2.4 and 281 m_osm_·kg^−1^, respectively (Table 1).

The mean value of VO_2_ max was 65 ± 4 mL·kg^−1^·min^−1^. The mean value of HR max was 187 ± 6 beats/min (Table 2).

In Phase A, a significant decrease (*p* < 0.001) was observed in many of the spirometry values before and after CPET. In more detail, FEV_1_ values after CPET were 4.9 ± 0.5 L (108.5 ± 8.4%), significantly lower than 5.2 ± 0.4 L (114.0 ± 6.9%) respective values before CPET. FVC values after CPET were 6.1 ± 0.6 L (112.4 ± 6.7%) significantly lower than the 6.4 ± 0.6 L (117.1 ± 6.7%) respective values before CPET. Similar findings were found for MEF_25–75_ values. After CPET, they were 4.4 ± 1.2 L (90.8 ± 11.5%), significantly lower than 5.0 ± 1.1 L (103.1 ± 8.3%) values before CPET (Table 3).

Applying the criteria for EIB [8], it was found that 30 (30%) cyclists have a reduction only in FEV_1_ ≥ 10%. Additionally, 33 (33.0%) participants had a reduction only in MEF_25–75_ ≥ 20%. Both criteria were true in 20 (20.0%) cyclists, while one of these criteria was true in 43 (43%) cyclists.

**Figure 1:**
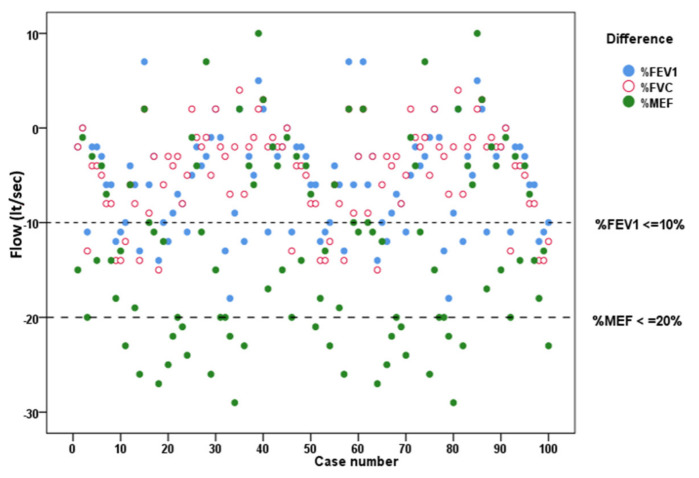
Spirometry differences in phase A of the study and the number of participants in phase B of the study (FEV_1_ = Forced Expiratory Volume in 1st sec, FVC = Forced Vital Capacity, MEF = Maximal Mild-Expiratory Flow Rate).

Forty-three (43%) athletes were eligible to continue in Phase B of the study. Two of the forty-three cyclists did not participate in Phase B for personal reasons. Finally, 41 (41%) athletes were included in Phase B (Table 4, Figure 1).

A comparison of the characteristics of athletes with and without a reduction in lung function is shown in Table 5. Cyclists who showed a reduction in lung function were significantly younger (24.0 ± 6.0 years) than those who did not show a reduction in lung function (29.0 ± 5.0 years, *p* < 0.001). The same was observed for training age (10.0 ± 6 vs. 14.0 ± 5.0 years, *p* < 0.001). Cyclists with a reduction in lung function were shorter than cyclists without a reduction in lung function (175.0 ± 6.0 vs. 179 ± 4.0 cm). No significant differences were found for BMI (*p* = 0.346) between participants with and without a reduction in lung function (23.9 ± 1.9 vs. 23.6 ± 1.0 kg/m^2^). Additionally, no significant differences were found for body fat (*p* = 0.352) between cyclists with and without a reduction in lung function (11.5 ± 0.9 vs. 11.7 ± 1.0%). Finally, hydration status (% of hydration) was significantly lower (*p* < 0.001) in cyclists with than without a reduction in lung function (46.0 ± 2.2 vs. 59.0 ± 4.7%). Additionally, P_osm_ was significantly higher (*p* = 0.016) in cyclists with than without a reduction in lung function (286.07 ± 1.2 vs. 282.08 ± 1.3 m_osm_·kg^−1^).

### 3.2. Phase B of the Study (Hydration Prior to Exercise)

The 41 cyclists who participated in Phase B (hydration phase), showed a difference in body hydration as expected from the experimental process (*p* < 0.001). More specifically, mean hydration in Phase B was 49.0 ± 2.3 vs. 46.0 ± 2.2% in Phase A of the study. The mean P_osm_ in Phase B was 280.1 ± 1.2 vs. 286.07 ± 1.2 m_osm_·kg^−1^ in Phase B. Additionally, the mean VO_2 max_ in Phase B was 64.1 ± 4.4 mL·kg^−1^·min^−1^, higher than the VO_2 max_ in Phase A (*p* < 0.001), which was 61.8 ± 4.3 mL·kg^−1^·min^−1^ (Table 6).

**Figure 2:**
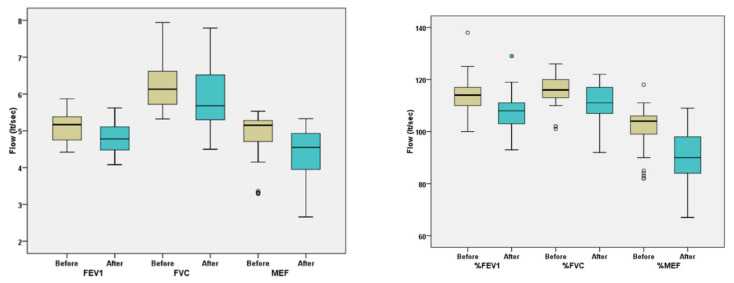
Differences in absolute (**left** ) and relative (**right** ) spirometry values before and after exercise in Phase B (FEV_1_ = forced expiratory volume in 1st s, FVC = forced vital capacity, MEF = maximal mild-expiratory flow rate). ^○^ *p* < 0.05.

In Phase B (hydration prior to exercise), the absolute and relative (%) values of FEV_1_, FVC, and MEF_25–75_ (Table 7, Figure 2) were statistically lower after CPET (*p* < 0.001). Based on relative values, FEV_1_ after CPET was 107.2 ± 7.7% vs. FEV_1_ before CPET 114.1 ± 7.6% (*p* < 0.001). FVC after CPET was 111.0 ± 7.9% and before CPET was 116.2 ± 6.2% (*p* < 0.001). Finally, MEF_25–75_ after CPET was 90.4 ± 11.0%, and before CPET was 101.7 ± 8.2% (*p* < 0.001).

The mean difference in the relative value of FEV_1_ in Phase B of the study was estimated at −6.9 ± 3.0% and was significantly lower (*p* < 0.001) than the difference in FEV_1_ in Phase A, which was -10.8 ± 2.7%. The mean difference in FVC was −5.2 ± 4.6% in Phase B and −8.3 ± 4.6 in Phase A (*p* < 0.001). Finally, the mean difference in MEF_25–75_ was −11.3 ± 7.6% in Phase B, and −20.7 ± 4.6 in Phase A (*p* < 0.001) (Table 8).

Applying the criteria for EIB [8], it was found that in Phase B 4 (9.7%) cyclists had a reduction in FEV_1_ ≥ 10% vs. 30 (30%) of the cyclists in Phase A. Additionally, 6 (14.6%) of the participants had a reduction in MEF_25–75_ ≥ 20% in Phase B vs. 33 (33.0%) of the participants in Phase A. Both criteria were true in 2 (4.8%) cyclists in Phase B vs. 20 (20%) cyclists in Phase A of the study, while one of these criteria was true in 6 (14.6%) cyclists in Phase B vs. 43 (43%) in phase A. The differences between phases A and B were statistically significant (*p* < 0.001).

A significant positive correlation was found between hydration and post-exercise FEV_1_ in Phase B of the study (r = 0.338, *p* = 0.031). Additionally, a significant correlation was found between hydration and VO_2_ max in Phase B (r = 0.449, *p* = 0.003). Finally, we found a positive correlation between post-exercise FEV_1_ and VO_2_ max, but this correlation was not statistically significant (r = 0.252, *p* = 0.112) (Table 9). 

## 4. Discussion

The findings of this study showed negative alterations in pulmonary function after exercise in elite cyclists. The observed reduction in spirometry values suggests that the reduction in pulmonary function is localized to all bronchial trees, i.e., in large and small airways. Exercise-induced alterations in pulmonary function were not observed at the same level when a systemic hydration protocol (via oral fluid intake) was applied before exercise. Systemic hydration may play a regulatory role in stabilizing bronchial tree potency and reducing bronchial hyperresponsiveness in athletes.

### 4.1. Effects of Exercise on Pulmonary Function

This study showed a reduction in pulmonary function after CPET among professional cyclists. These findings are in line with a previous study that showed an acute reduction in FEV_1_ after prolonged exercise among recreational runners [47]. This study states that the prolonged endurance exercise reduced lung function by ~20% in FEV_1_ and EIB is the most probable explanation for this reduction. In this study, EIB did not affect the finish time among participants. Another study reported that exercise is the most common trigger of bronchoconstriction in non-professional non-asthmatic marathoners and half marathoners [48]. This study showed a decline in FEV_1_ ≥10% in 35.29% of marathon and 22.22% of half marathon runners. In another study [49], which includes fifty-eight elite runners, it was found that a post-exercise reduction of 10% or more in FEV_1_ was observed in five (9%) of the 58 runners. In the same study, it was also supported that the occurrence of EIB is common in elite runners and is strongly associated with atopy (positive skin prick test reactions). Finally, exercise was not performed under laboratory conditions with constant temperature and humidity levels. In our study, athletes with laboratory-confirmed atopy (IgE-positive) were not included and the exercise was performed under laboratory conditions. Our results from a healthy population of professional cyclists suggest that exercise-induced pulmonary impairment is a general phenomenon that may be present irrespective of the presence of pulmonary disease. Another study reported improvements in pulmonary function following moderate non-exercise-induced dehydration (i.e., 4.0–to–4.5% loss of body mass) induced by diuretics in healthy men [22]. In this study, the lung volumes increased significantly. In addition, lung function test results, including FEV_1_, increased significantly and returned to normal upon rehydration. The improvement in ventilatory lung function during hypohydration was surprising, and it is suggested that this was related to the loss of water within and/or around the airways [22]. However, the use of diuretics could explain the divergent findings, as diuretics cause iso-osmotic hypovolemia, whereas exercise led to hyperosmotic hypovolemia [50]. This study examined the physiological basis for understanding dehydration and highlight how interpretations of dehydration depend on the type (dehydration compared with volume depletion) and severity (moderate compared with severe) of dehydration, which in turn influences the plasma osmolality.

### 4.2. Effects of Hydration on Pulmonary Function 

A novel finding of the current study is that systemic hydration is effective in preventing reduction in pulmonary function after exercise in professional athletes. Therefore, our findings suggest that oral fluid intake before exercise is an effective strategy to prevent exercise-induced pulmonary alterations and can lead to a reduction in the development of EIB among non-asthmatic and non-atopic professional cyclists.

A previous study supported that systemic rehydration restores dehydration-induced changes in pulmonary function in healthy adults [51]. This study aimed to clarify the impact of exercise-induced dehydration or fluid restriction-induced dehydration on pulmonary function in healthy adults and establish whether systemic or local rehydration can reverse dehydration-induced alterations in pulmonary function. Ten (10) healthy participants performed four experimental trials in a randomized order (2 h exercise in the heat twice and 28 h fluid restriction twice). Pulmonary function was assessed using spirometry in the hydrated, dehydrated, and rehydrated states. Oral fluid consumption was used for systemic rehydration and nebulized isotonic saline inhalation for local rehydration. Both exercise and fluid restriction induced mild dehydration and elevated plasma osmolality. Dehydration was accompanied by a reduction in FVC, with no statistical differences between the dehydration modes. These changes were normalized by fluid consumption but not by nebulization. The results of the study suggest that, in healthy adults, mild systemic dehydration induced by exercise or fluid restriction leads to pulmonary function impairment, primarily localized to small airways, and systemic, but not local, rehydration reverses these potentially harmful alterations. This study demonstrates that, in healthy adults, dehydration induced by exercise in the heat leads to negative alterations in pulmonary function, primarily localized to small airways. Oral rehydration can restore pulmonary function in dehydrated individuals. Another study reported an inverse correlation between increased serum osmolality and decreased FVC and FEV_1_ in a large population of patients with chronic obstructive pulmonary disease COPD [52]. This study aimed to evaluate the hypothesis that increased serum osmolality is associated with lower FEV_1_ and FVC values. Increased serum osmolality was inversely associated with both FEV_1_ and FVC values. This study suggested a causal relationship between increased serum osmolality and reduced pulmonary function. 

It is possible that oral fluid consumption might have led to psychological benefits, and thereby, improving the effort during pulmonary function tests. Indeed, mood has been shown to improve after rehydration with oral fluid [53]. However, the literature mentions that too much fluid ingestion while exercising could induce pulmonary edema [54,55].

The MEF_25–75_% is a potentially sensitive marker of obstruction of peripheral airflow [56], and it is reduced in early bronchial impairment, which is associated with small airway disease [57]. The role of small airways in EIB still needs to be clarified and more studies are needed to assess if small airway function and inflammation may be considered reliable predictive markers of EIB in elite athletes [58]. In our study, there is a strong correlation between hydration status and improvement in MEF_25–75_% among elite cyclists.

Endurance athletes are at increased risk for exercise-induced dehydration and commonly report respiratory symptoms while exercising [59]. Diagnosis is relevant because of its potential implications on the performance of elite athletes [59]. In contrast, untreated or undertreated respiratory symptoms result in chronic inflammation associated with epithelial damage, which contributes to airway remodeling and fibrotic changes, fixed obstruction, and progressive decline in lung function over time [59].

### 4.3. Effects of Hydration on Exercise Capacity

Hydration plays a significant role in athletic performance [60], including its impact on maximal oxygen uptake (VO_2_ max). VO_2_ max is a measure of an individual’s maximum capacity to utilize oxygen during intense exercise. Proper hydration helps regulate body temperature during exercise [57,58]. When the body is dehydrated, it struggles to dissipate heat efficiently, leading to an increase in core body temperature [61]. Elevated body temperature can negatively affect VO_2_ max [61,62]. Adequate hydration supports thermoregulation, allowing athletes to maintain optimal body temperature and, in turn, preserve their VO_2_ max [60,61,62]. Hydration status affects blood volume [63], which is crucial for oxygen delivery to working muscles [64]. Additionally, dehydration reduces blood volume [63] and increases blood viscosity [65]. As a result, oxygen delivery to the muscles decreases, limiting the VO_2_ max. Optimal hydration helps maintain proper blood volume, promoting efficient oxygen transport and utilization. Finally, dehydration can lead to an elevated heart rate [63,66] and increased cardiovascular strain [63,67]. A higher heart rate can reduce cardiac output during exercise, leading to reduce the oxygen delivery to the muscles, affecting VO_2_ max. Maintaining hydration levels helps regulate heart rate and cardiac output, supporting optimal VO_2_ max. Our study is in accordance with the literature, supporting a strong correlation between hydration status and improvement in VO_2_ max among elite cyclists.

The major limitation of this study is that we did not monitor water loss for each participant during training. Consequently, it remains unclear whether the daily consumption of 3 L of water is sufficient to ensure hydration on hard training days. In addition, we considered as a limitation of the study that all participants were males. The menstrual cycle and female hormonal fluctuations were the only reasons why females were not included in this study. Exercise performance may be reduced during the menstrual cycle. Finally, MEF_25–75_% is widely used to estimate small airway dysfunction, but it has some limitations. Apart from spirometry, there are many other methods for assessing small airway function, such as impulse oscillometry, computed tomography, body plethysmography, inert gas washout, and Magnetic Resonance Imaging. 

## 5. Conclusions

The findings of the study support that elite athletes may have non-beneficial effects on respiratory function. Systemic hydration has a positive effect on both VO_2_ max and pulmonary function in elite athletes. Therefore, hydration plays a regulatory role in stabilizing the airway in elite athletes and protecting them from airway hyper-responsiveness. Of particular interest are the small airways, which appear to be affected independently or in combination with the decrease in FEV_1_ and the proper hydration can protect them from further injury. The role of small airways in EIB still needs to be clarified and more studies are needed to assess if small airway function and inflammation may be considered reliable predictive markers of EIB in elite athletes.

A novel finding of the current study is that systemic hydration is effective at preventing reduction in pulmonary function after exercise in elite athletes. Our findings suggest that oral fluid intake before exercise is an effective strategy to prevent exercise-induced pulmonary alterations and can lead to a reduction in the development of EIB among non-asthmatic and non-atopic professional cyclists.

## Figures and Tables

**Table 1:** Descriptive statistics of age, training age, and somatometric variables. Data are expressed as percent, mean ± SD.

		Mean ± SD	m (IQR)	Min–Max
Age	years	27.0 ± 5.0	30 (22–33)	18–34
Training age	years	12.0 ± 5.0	14 (7–17)	3–19
Height	cm	177 ± 5	178 (174–182)	167–187
Body mass	kg	74.7 ± 5.2	74.6 (72–78)	64–85
BMI	kg/m^2^	23.8 ± 1.4	23.6 (22.6–24.4)	21.5–26.8
Body fat	%	11.6 ± 1.0	11.7 (11–12.4)	11.3–13.1
Hydration	%	53.0 ± 7.0	53.0 (46.0–58.0)	41.0–69.0
P_osm_	m_osm_·kg^−1^	283 ± 2.4	281 (279–286)	278–288

**Table 2:** Baseline of oxygen uptake (VO_2_) and heart rate (HR) before hydration. Data are expressed as percent, mean ± SD.

		Mean ± SD	m (IQR)	Min–Max
VO_2_ max	mL·kg^−1^·min^−1^	65 ± 4	62 (60–65)	54–74
HR max	bpm	187 ± 6	186 (183–190)	180–206

m (IQR) = Median (Interquartile range).

**Table 3:** Absolute and relative (%) spirometry values before and after CPET in Phase A of the study. Data are expressed as percent, mean ± standard deviation.

		Phase I		*p* Values
		Before CPET	After CPET	
		Mean ± SD	Mean ± SD	
FEV_1_	L	5.2 ± 0.4	4.9 ± 0.5	<0.001
	% of pred.	114.0 ± 6.9	108.5 ± 8.4	<0.001
FVC	L	6.4 ± 0.6	6.1 ± 0.7	<0.001
	% of pred.	117.1 ± 6.7	112.4 ± 8.9	<0.001
MEF_25–75_	L	5.0 ± 1.1	4.4 ± 1.2	<0.001
	% of pred.	103.1 ± 8.3	90.8 ± 11.5	<0.001

**Table 4:** Spirometry differences in Phase A of the study before and after CPET and the number of participants in Phase B.

Differences in Percentages		*n*	%
FEV_1_	<10%	70	70
	≥10%	30	30
MEF_25–75_	<20%	67	67
	≥20%	33	33
Both criteria		20	20
One of these criteria		43	43
Phase B		41	41

FEV_1_ = forced expiratory volume in 1st sec, MEF = maximal mild-expiratory flow rate.

**Table 5:** Differences in characteristics between athletes with and without reduction in lung function in Phase A.

		Reduction in Lung Function		
		No (*n* = 59)	Yes (*n* = 41)	*p* Value
		Mean ± SD	Mean ± SD	
Age	Year	29.0 ± 5.0	24.0 ± 6.0	<0.001
Training age	years	14.0 ± 5.0	10.0 ± 6.0	<0.001
Height	cm	179 ± 4.0	175 ± 6.0	0.001
Body mass	kg	75.6 ± 4.1	73.5 ± 6.4	0.047
Body mass index	kg/m^2^	23.6 ± 1.0	23.9 ± 1.9	0.346
Body fat	%	11.7 ± 1.0	12.5 ± 0.9	0.352
Hydration	%	59.0 ± 4.7	46.0 ± 2.2	<0.001
P_osm_	m_osm_·kg^−1^	282.08 ± 1.3	286.07 ± 1.2	0.016

Data are expressed as percent, mean ± SD.

**Table 6:** Differences in 41 participants between Phase A and Phase B.

		Phase A	Phase Β	
		Mean ± Sd	Mean ± Sd	*p* Values
BMI	kg/m^2^	23.9 ± 1.9	23.9 ± 1.9	0.130
Body fat	%	21.5 ± 0.9	21.6 ± 0.9	0.210
Hydration	%	46.0 ± 2.2	49.4 ± 2.3	<0.001
VO_2_ max	mL·kg^−1^·min^−1^	61.8 ± 4.3	64.1 ± 4.4	<0.001
P_osm_	m_osm_·kg^−1^	286.07 ± 1.2	280.1 ± 1.2	<0.001

**Table 7:** Comparison of spirometric values in Phase B before and after CPET. Data are expressed as percent, mean ± SD.

Phase B				
		Before CPET	After CPET	
		Mean ± SD	Mean ± SD	*p* Values
FEV_1_	L	5.1 ± 0.4	4.8 ± 0.4	<0.001
	% of pred.	114.1 ± 7.6	107.2 ± 7.7	<0.001
FVC	L	6.2 ± 0.7	5.9 ± 0.8	<0.001
	% of pred.	116.2 ± 6.2	111 ± 7.9	<0.001
MEF_25–75_	L	4.9 ± 0.7	4.4 ± 0.7	<0.001
	% of pred.	101.7 ± 8.2	90.4 ± 11	<0.001

**Table 8:** The mean differences in relative values in Phase A and B of the study. Data are expressed as percent, mean ± SD.

	Phase A	Phase B	*p* Values
% Differences	Mean ± SD	Mean ± SD	
FEV_1_	−10.8 ± 2.7	−6.9 ± 3	<0.001
FVC	−8.3 ± 4.6	−5.2 ± 4.6	<0.001
MEF	−20.7 ± 4.6	−11.3 ± 7.6	<0.001

FEV_1_ = forced expiratory volume in 1st s, FVC = forced vital capacity, MEF = maximal mild-expiratory flow rate.

**Table 9:** Correlation between Hydration status, VO_2_ max, and FEV_1_ in Phase B.

		FEV_1_	VO_2_ max
HYD%	Pearson’s R correlation	0.449	0.338
	Sig. (2-taled)	0.003	0.031

## Data Availability

The data presented in this study are available on request from the corresponding author.
